# Cationic Tantalum Oxide Nanoparticle and Neutral Iodixanol Contrast Agents Alter Cartilage Mechanical Properties

**DOI:** 10.1002/jor.70297

**Published:** 2026-10-19

**Authors:** Maria Rissanen, Jiri Jäntti, Juuso Tuppurainen, Milka Poimala, Brian D. Snyder, Mark W. Grinstaff, Mika E. Mononen, Miitu K. M. Honkanen, Janne T. A. Mäkelä

**Affiliations:** ^1^ Department of Technical Physics University of Eastern Finland Kuopio Finland; ^2^ Diagnostic Imaging Center Kuopio University Hospital, Wellbeing Services County of North Savo Kuopio Finland; ^3^ Boston Children's Hospital Boston Massachusetts USA; ^4^ Departments of Biomedical Engineering Chemistry, and Medicine, Boston University Boston Massachusetts USA; ^5^ Center of Oncology Kuopio University Hospital, Wellbeing Services County of North Savo Kuopio Finland

**Keywords:** articular cartilage, biomechanics, contrast agents, finite element modeling, microcomputed tomography

## Abstract

Early detection of cartilage degeneration remains a critical unmet clinical need. While contrast‐enhanced computed tomography shows promise in detecting early structural and compositional changes in articular cartilage, full adoption into clinical use requires a comprehensive understanding of how contrast agents affect cartilage function, including potential biomechanical alterations. This study investigates how exposure to two contrast agents, pre‐clinical ultrasmall cationic tantalum oxide nanoparticles (Ta_2_O_5_‐cNPs) and neutral iodixanol, approved for clinical use, affect the mechanical and frictional properties of articular cartilage. Biomechanical and tribological properties of osteochondral samples (*n* = 60) from adult bovine stifle joints (*N* = 9) were systematically assessed before and after contrast agent immersion using friction testing, stress‐relaxation, and dynamic loading protocols. The effect of solid and charged contrast agents on extracellular matrix stiffness and fluid‐flow properties was studied using finite element modeling, which enabled interpretation of the stress‐relaxation response and extraction of constituent‐specific mechanical parameters. The results demonstrate that Ta_2_O_5_‐cNP uptake softens extracellular matrix of cartilage as non‐fibrillar matrix modulus decreases (linear mixed model, *p* < 0.001). Both contrast agents reduce permeability and viscosity of cartilage. These findings highlight the sensitivity of articular cartilage mechanics to contrast‐agent uptake and distribution within the tissue. The results underscore the importance of carefully considering nanoparticle charge and size together with other physicochemical properties, such as composition, concentration, surface chemistry, and tissue retention, when designing contrast agents for cartilage imaging or therapy.

## 1 Introduction

The mechanical response of articular cartilage (AC) to loading is governed in part by the interaction between proteoglycans (PGs) and interstitial fluid, together with collagen network. Negative charge of PGs causes electrostatic repulsion forces and fixed charge density (FCD) inside the cartilage, which contributes to matrix stiffness, drives osmotic swelling, and contributes to interstitial fluid pressurization under loading [1]. This pressurization promotes fluid film lubrication in synovial joint, ensuring low friction and minimal wear of healthy cartilage [2, 3]. The collagen network preserves structural conformation and resists tensile forces through its tensile strength and cross‐linking [4, 5]. In early osteoarthritis (OA), cartilage undergoes microscopic structural changes, leading to increased permeability and swelling, decreasing its mechanical stiffness and causing increased friction and wearing [6, 7, 8]. Current OA diagnostic approaches, such as the Kellgren‐Lawrence classification, rely on plain radiography in addition to clinical examination and symptoms described by patient [9]. They are effective for identifying later‐stage joint damage but lack the sensitivity to detect early alterations in the AC, including PG and collagen degradation [10].

Conventional clinical contrast‐enhanced computed tomography (CECT) has primarily relied on iodinated contrast agents [11]. Beyond clinical use, CECT offers high‐resolution imaging of cartilage morphology and composition [12, 13, 14]. More recently, metallic‐core nanoparticles (NPs) have emerged as a promising new class of contrast agents, offering tunable physicochemical properties [15]. Their size and surface charge can be tailored, enabling various cartilage imaging applications, such as segmentation and detection of surface injuries [16, 17, 18]. Additionally, the dense core attenuates X‐rays efficiently, and they have higher *k*‐edge energies, at which X‐ray attenuation increases sharply, enabling stronger contrast at higher X‐ray energies compared to conventional iodinated contrast agents [16, 19, 20]. However, for effective intra‐cartilage diffusion, NPs must remain ultrasmall and typically below 6 nm in diameter [1]. Among potential NP candidates, ultrasmall (~3 nm) cationic tantalum oxide nanoparticles (Ta_2_O_5_‐cNPs) have shown promise in soft tissue imaging due to their high attenuation efficiency and ability to electrostatically bind to the negatively charged PGs [20, 21]. Jäntti et al. [20] demonstrated that the uptake of Ta_2_O_5_‐cNPs in cartilage correlates with depth‐wise PG distribution, collagen content, and equilibrium modulus. In addition, tuning the nanoparticle surface charge allowed segmentation of cartilage lesions [22].

Although CECT has potential for early OA detection and assessment of cartilage composition, the effects of contrast agents on cartilage mechanical properties remain unclear. Berni et al. [23] demonstrated that the cationic iodine‐based contrast agent CA4+ softens cartilage by reducing both its instantaneous and equilibrium stiffness. However, the mechanistic basis of such alterations, whether due to fixed charge neutralization, structural disruption, or fluid flow interference, remains poorly understood. To overcome these limitations, computational modeling approaches, such as fibril‐reinforced biphasic models, can be used to disentangle the contributions of cartilage constituents [24, 25, 26, 27, 28, 29]. As previously employed, fibril‐reinforced poroelastic (FRPE) material model defines cartilage behavior with five parameters: non‐fibrillar matrix modulus describes mechanical effect of PGs and governs equilibrium stiffness and resilience; fibril network moduli reflect initial stiffness of collagen and its nonlinear strain stiffening; initial permeability and its strain‐dependency factor regulate fluid flow, influencing load bearing and hydration [28, 30]. Conventional biomechanical testing alone cannot separate these constituent‐specific contributions to the overall tissue response, underscoring the value of simulation‐based interpretation. The cartilage softening observed by Berni et al. [23] with cationic molecular iodine‐based contrast agent raises the question of whether metallic NPs, especially those with cationic surface charge, exert similar or opposite effects on cartilage biomechanics. Due to their dense solid core, metallic NPs could hypothetically reinforce the cartilage matrix and increase stiffness. Moreover, if retained at the surface, NPs may reduce cartilage‐on‐cartilage contact by forming a physical barrier, thus lowering friction and potentially slowing tissue wear [31, 32]. This concept of dual‐function has been recently explored by Lawson et al. [33], who introduced cationic dual‐purpose Ta_2_O_5_‐NPs (hydrodynamic diameter ~215 nm) that simultaneously serve as imaging agent and joint lubricant in osteoarthritic models. However, the effect of diffusing ultrasmall (< 6 nm) nanoparticles on the biomechanics and frictional properties of healthy cartilage remains unknown.

In this study, we examine the effect of cationic Ta_2_O_5_‐NP contrast agent on the biomechanical properties of articular cartilage, including friction, constituent‐specific mechanics, and viscoelastic behavior. For reference, we also examine the effect of neutral iodixanol contrast agent, which is approved for clinical use. We hypothesize that (1) metal‐core Ta_2_O_5_‐cNPs reinforce the extracellular matrix of cartilage; (2) Ta_2_O_5_‐cNPs decrease friction between cartilage and glass surfaces; and (3) due to its neutral charge and small size, iodixanol has a minimal effect on cartilage biomechanics. To address our hypotheses, we conducted friction testing following by stress‐relaxation and dynamic mechanical testing, both before and after contrast agent immersion, using osteochondral samples harvested from adult bovines. Stress‐relaxation testing was simulated with finite element (FE) modeling to estimate changes in matrix‐, fibril‐, and fluid‐related mechanical behavior. This approach enables interpretation of how contrast agents influence the relative contributions of these mechanisms to cartilage load bearing and lubrication.

## 2 Materials and Methods

### 2.1 Samples

Cylindrical osteochondral samples (*d* = 7 mm) were harvested from the knee joints of adult bovines (*N* = 9, acquired from Atria Suomi Oy, Atria) from two anatomical locations: the medial femoral condyle (MFC, *n* = 31) and the distal intertrochlear groove (DITG, *n* = 29) (Figure 1). The samples were stored at −20°C in phosphate‐buffered saline (PBS, pH 7.4, osmolality 310 mOsm/kg), which contained protease inhibitors [5 mM, ethylenediamine‐tetra‐acetic acid disodium salt dihydrate (EDTA; VWR International, Radnor, PA, USA) and 5 mM benzamidine hydrochloride hydrate (Sigma‐Aldrich Co., St. Louis, MO, USA)].

**Figure 1:**
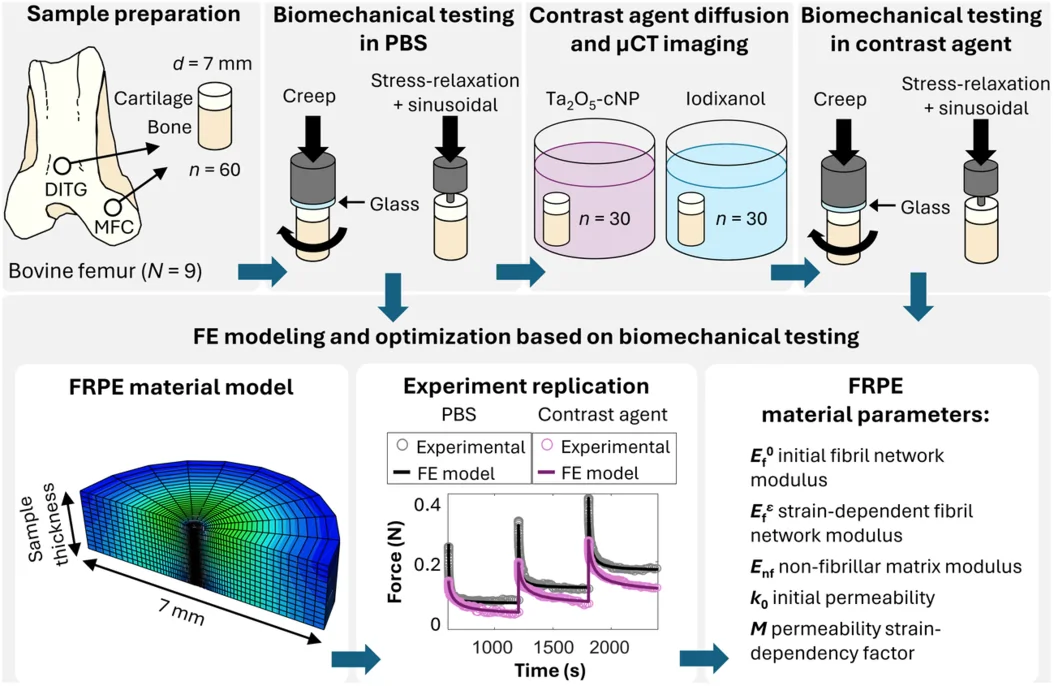
Workflow of the study: Bovine osteochondral samples (*n* = 60) were harvested from nine femurs at two anatomical locations: the medial femoral condyle (MFC, *n* = 31) and the distal intertrochlear groove (DITG, *n* = 29). Biomechanical testing (unconfined friction and indentation) was first performed in phosphate‐buffered saline (PBS). Samples were then divided between cationic tantalum oxide nanoparticle (Ta_2_O_5_‐cNP) and neutral iodixanol contrast agent baths (*n* = 30 each). Samples were imaged with microcomputed tomography (µCT) at multiple time points to monitor diffusion up to 93 h (Ta_2_O_5_‐cNP) and 38 h (iodixanol). After equilibration, biomechanical testing was repeated in the respective contrast agents. Stress‐relaxation tests were replicated with an axisymmetric fibril‐reinforced poroelastic (FRPE) finite element (FE) model (visualized in 3D for clarity) to estimate sample‐specific material parameters.

### 2.2 Biomechanical Testing

After thawing the samples at room temperature, the cartilage thickness was determined from microcomputed tomography (μCT, Nikon XT H 225, Nikon Metrology Europe, Leuven, Belgium) images acquired with an isotropic voxel size of 40 μm, and analyzed using a custom‐made MATLAB script (R2022b, Mathworks, Natick, MA, USA). Friction and indentation testing (Figure 1) were first conducted in PBS and repeated after contrast agent immersion (93 h for Ta_2_O_5_‐cNP and 38 h for iodixanol). The repeated testing was conducted in the same contrast agent bath in which the sample was immersed. The friction and indentation measuring setups comprised a 0.1 μM resolution displacement actuator (PM1A11939, Newport, Irvine, CA, USA) and a 10 kg and 0.25 kg load cell (Model 31, Honeywell International Inc., Charlotte, NC, USA), respectively. The samples were fixed to a sample holder from the subchondral bone with three screws and the holder was filled with medium (PBS or contrast agent). In indentation geometry, the sample holder was fixed to a goniometer to improve the visual assessment of surface perpendicularity between the cartilage and the indenter. The friction‐measuring geometry had an additional torque sensor (Model TFF400, Futek, Irvine, CA, USA) and a rotating motor (Oriental Motor, Tokyo, Japan) to which the sample holder was fixed.

Friction was measured using a rotational geometry, with a cartilage plug rotating against a glass surface under unconfined compression [34]. Contact was first ensured with 12.5 kPa compressive stress, after which samples were pre‐conditioned with 10 sinusoidal load‐unload cycles to 5% of cartilage thickness to stabilize the mechanical response and promote fluid redistribution within the tissue, followed by a 10‐min recovery. Friction testing was then performed by compressing cartilage to 0.05 MPa (1.92 N) at 0.05 mm/s, with the plug rotating at 1.19 rev/s against the glass. After 30 s of rotation, the glass was lifted 100 µM for 1 s to allow fluid re‐entry before reversing rotation, generating a 20‐step protocol. The coefficient of friction (CoF) was calculated as follows [2, 35]:

(1) $$\mathrm{CoF}=\frac{3}{2}\frac{T}{\mathrm{Nr}},$$

where T is the measured torque, N is the measured compressive force, and r is the radius of the sample. Initial CoF was calculated as the mean of the CoF value when 0.05 MPa compression was reached during the second and third step, and equilibrium CoF as the mean of the CoF value at the end of the second last and last cycle [34]. The initial and equilibrium CoF were analyzed as the mean CoF during one rotation cycle (0.84 s).

After the friction test, sample was transferred to the indentation measuring setup and let to recover for 10 min. Four‐step stress‐relaxation protocol was conducted with a plane‐ended cylindrical indenter (*d* = 0.55 mm), as previously described [36]. Full contact between the indenter and cartilage surfaces was ensured with a pre‐stress of 40 kPa, followed by a 10‐min relaxation. The stress‐relaxation steps utilized a 4% step size, 100%/s ramp rate, and 10‐min relaxation. Then, continuing from the final indentation depth, a dynamic test followed, using a four‐cycle sinusoidal protocol with 4% peak‐to‐peak strain amplitude at 1 Hz, as previously described [30]. Phase difference (∆*θ*) was determined from the dynamic test [37], to investigate the viscoelastic behavior of cartilage.

### 2.3 Finite Element Modeling

Sample‐specific axisymmetric FRPE FE models were constructed (Figure 1). See the Supporting Information for details on the material model and finite element analysis. Briefly, the fibrillar phase was modeled as a tension‐only with nonlinear collagen network with strain‐dependent stiffening, the non‐fibrillar solid matrix as Neo‐Hookean hyperelastic material carrying all compressive stresses, and the fluid phase as interstitial flow governed by Darcy's law with strain‐dependent permeability [38, 39].

To replicate the experimental stress‐relaxation tests, the following FRPE model parameters were optimized: initial fibril network modulus ($E_{f}^{0}$), strain‐dependent fibril network modulus ($E_{f}^{\varepsilon }$), non‐fibrillar matrix modulus ($E_{\text{nf}}$), initial permeability ($k_{0}$), and permeability strain‐dependency factor (M). These were obtained for each sample using MATLAB's *fminsearch* function by minimizing the mean‐squared relative error between the force‐time response of the model and the corresponding experimental stress‐relaxation test (excluding the first step) [30, 40, 41]. Initial cartilage thicknesses determined from μCT images were used for all models, as no difference in the cartilage thickness was observed after contrast agent immersion. Interstitial fluid load support (IFLS) was calculated from surface nodes in contact with the indenter as the ratio of the average pore pressure (POR) to the total contact pressure (CPRESS).

Additional FE simulations were created using the mean of material parameter values of each group (Table S1) to demonstrate the differences in the mechanical behavior of cartilage before and after contrast agent immersions. Mean values for the PBS model were calculated from all 60 samples. For the additional mean models, we applied uniform thickness and a fixed 4% displacement. The thickness was determined as the mean thickness across all samples prior to contrast agent immersions.

### 2.4 Contrast Agent Preparation and Diffusion Experiments

Ta_2_O_5_‐cNPs were synthesized as previously described [21]. As earlier, the Ta_2_O_5_‐cNP fraction used in this study was obtained after 0.45 µm filtration using an H‐PTFE filter (Whatman Puradisc syringe filters, Cytiva, Marlborough, MA, USA), followed by dialysis with a 3.5 kDa molecular weight cut‐off membrane (SnakeSkin dialysis tubing, Thermo Fisher Scientific, Waltham, MA, USA). The size of the particles was ~3 nm, measured using transmission electron microscopy [19]. The zeta potential of the Ta_2_O_5_‐cNPs was 35.01 mV, measured using electrophoretic light scattering (Zetasizer, Nano ZS, Malvern). The Ta_2_O_5_‐cNP contrast agent bath was prepared as previously described [20]. Neutral iodixanol contrast agent was commercial (320 mg·I/mL, Visipaque, GE Healthcare AS, Oslo, Norway) and purchased. For both contrast agents, the pH was adjusted to 7.4, and osmolality to 310 mOsm/kg. The osmolality was quantified using a freezing point osmometer (Halbmikro‐osmometer, GWB, KNAUER Wissenschaftliche Geräte GmbH, Berlin, Germany). The concentration of Ta_2_O_5_‐cNP contrast agent bath was set at 30 mg/mL, as previously [20], and the concentration of iodixanol bath was set at 160 mg·I/mL to approximate comparable X‐ray attenuation in cartilage. To minimize the dilution effects, the volume of the contrast agent bath was set to 6.16 mL per sample, corresponding to eighty times the estimated volume of the cartilage.

30 samples (MFC *n* = 15, DITG *n* = 15) were immersed in Ta_2_O_5_‐cNP bath for 93 h, and 30 samples (MFC *n* = 16, DITG *n* = 14) were immersed in iodixanol bath for 38 h at room temperature (Figure 1), and shaken gently with a three‐dimensional gyroscopic motion generator (Stuart Scientific Gyro rocker, STR9, Staffordshire, UK) during immersion. No sealing was applied, so contrast agents were free to diffuse into the cartilage from all sides of the sample, including the cut sides of the osteochondral plugs. An additional control set of osteochondral samples (*n* = 5) was immersed in PBS containing protease inhibitors in room temperature shaken gently during immersion for 93 h to assess whether prolonged immersion itself could affect cartilage mechanics. Indentation testing was conducted before and after 93 h period, and no measurable changes in any of the cartilage biomechanical properties were observed.

### 2.5 μCT Imaging of Cartilage and Image Analysis

Before and during contrast agent immersions, μCT imaging was conducted in air with the samples sealed in a plastic container and fixed to a styrofoam holder at 0, 3, 6, 24, 48, 72, and 93 h for Ta_2_O_5_‐cNP, and at 0, 2, 4, 15, 24, and 38 h for iodixanol. All images were acquired using a tube voltage of 140 kVp and a 0.5 mm copper filter. During the approximately 8 min 30 s scan time, five samples per scan were placed in a single plane and rotated full circle (360°), and 1440 frames were imaged. Isotropic voxel size was 40 µm.

Image analysis was conducted using a custom‐made MATLAB script (R2022b, Mathworks, Natick, MA, USA). The image stack was rotated in the x‐ and y‐ directions to align the cartilage surface horizontally. The air‐cartilage and cartilage‐bone interfaces were detected based on X‐ray attenuation thresholds at the center of the sample. Attenuation was analyzed from the center of the sample with a region‐of‐interest of 5.6 mm in diameter (relative volume‐of‐interest of 64%), subtracting native cartilage attenuation (0 h imaging). Contrast agent partition was then calculated by dividing the resulting value with the contrast agent attenuation of the corresponding contrast agent bath. Native contrast agent baths were imaged separately. An exponential function was fitted to the partition data of each sample to characterize the diffusion kinetics [20]:

(2) $$P(t)=P_{\max}\left(1-\text{exp}\left(-t/\tau \right)\right),$$

where $P_{\max}$ represents the maximum partition of the contrast agent at diffusion equilibrium, t is the diffusion time, and τ is the diffusion time constant, *i.e*., the time during which 63.2% of the maximum partition of contrast agent is reached.

### 2.6 Statistical Analysis

Comparisons of the mechanical parameters measured before and after the contrast agent immersions were conducted using a linear mixed model using IBM SPSS Statistics (version 29.0.2.2 (20)), IBM Corp., USA). The medium in which the mechanical testing was conducted was set as a fixed variable and the animal as a random subject variable. Differences were considered statistically significant at *p* < 0.05.

## 3 Results

### 3.1 Indentation Testing and Finite Element Modeling

The FE models accurately replicated experimental stress‐relaxation data, as the mean $R^{2}$ values and corresponding 95% confidence intervals (CIs) for each experiment were 0.94 (95% CI: 0.93, 0.96), 0.93 (95% CI: 0.92, 0.94) and 0.93 (95% CI: 0.90, 0.95) for PBS, Ta_2_O_5_‐cNP and iodixanol, respectively (Figure 2). Results of the FE model parameter and IFLS group means and corresponding 95% CIs are presented in detail in Supporting Information (Tables S1–S3). Significant differences in multiple optimized model parameters and viscoelasticity were observed after both contrast agent immersions (Figure 3). Non‐fibrillar matrix modulus $E_{\text{nf}}$, initial permeability $k_{0}$, and permeability strain‐dependency factor M were found to be lower (*p* < 0.001, Figure 3c–e) after Ta_2_O_5_‐cNP immersion. After iodixanol immersion, $k_{0}$ and M were found to be lower (*p* < 0.001, Figure 3d,e). Phase difference ∆θ, which describes the viscoelastic behavior of cartilage, decreased after Ta_2_O_5_‐cNP and iodixanol immersions (*p* < 0.001 and *p* = 0.016, respectively, Figure 3f). The differences in initial and strain‐dependent fibril network moduli were not significant (Figure 3a,b). The relationships of non‐fibrillar matrix modulus and initial permeability before and after Ta_2_O_5_‐cNP were $E_{\text{nf}}:\;0.259x+0.038$, and $k_{0}:\;0.120x+0.076$, respectively (Figure 4a,b). Similarly, the relationship of initial permeability before and after iodixanol was $k_{0}:\;0.074x+0.235$ (Figure 4b).

**Figure 2:**
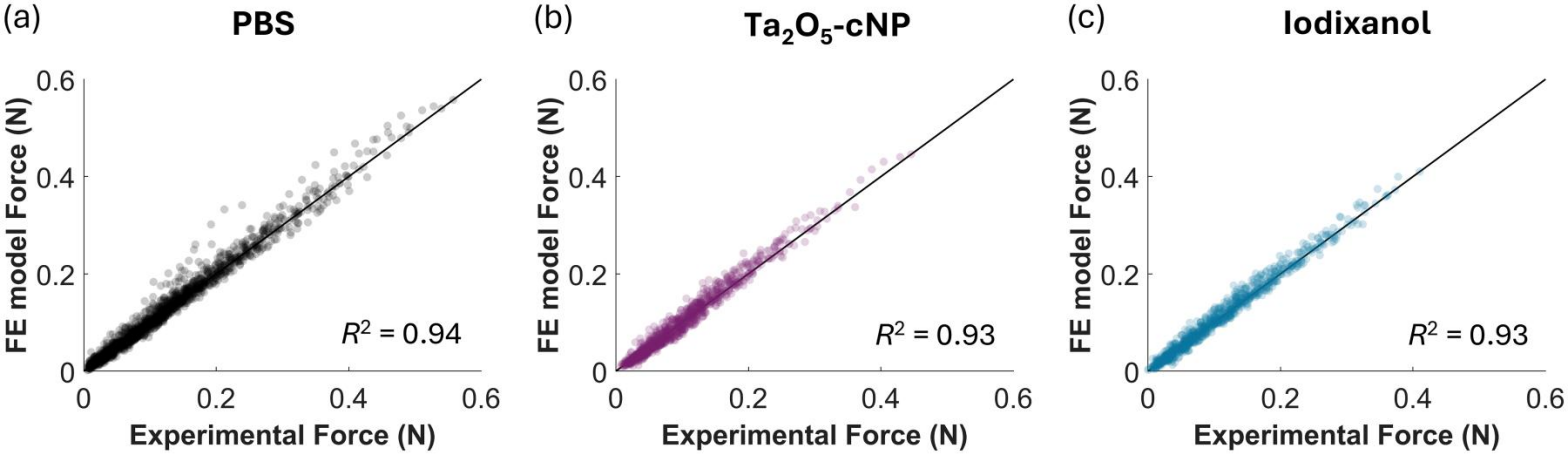
Experimentally measured force during stress‐relaxation test to corresponding force from the finite element (FE) model. Parameter values are shown for samples measured in (a) phosphate‐buffered saline (PBS), (b) cationic tantalum oxide nanoparticle (Ta_2_O_5_‐cNP) contrast agent, and (c) iodixanol contrast agent. Straight line represents the one‐to‐one ratio. The mean *R* ^2^ quantifying model fitting to experimental data. 10 data points per relaxation step were utilized for this figure.

**Figure 3:**
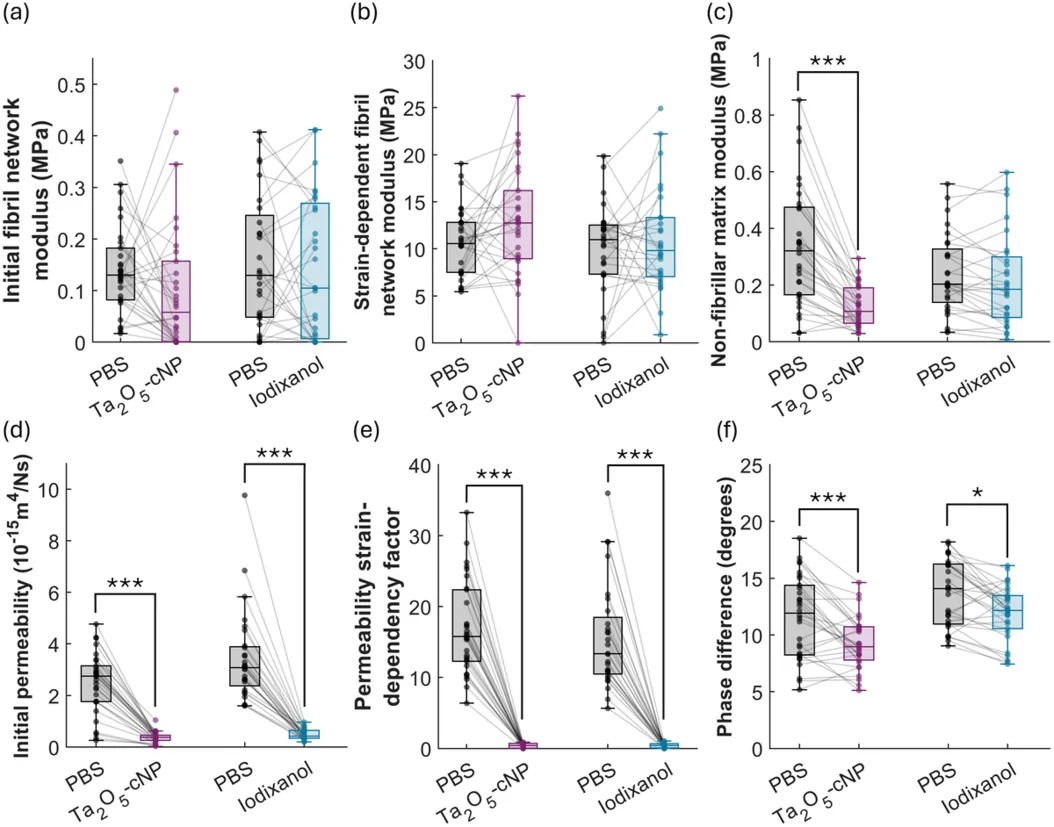
Constituent‐specific finite element (FE) model parameters and viscoelastic behavior of samples before (measured in phosphate‐buffered saline [PBS]) and after cationic tantalum oxide nanoparticle (Ta_2_O_5_‐cNP) and iodixanol immersion. (a) Initial fibril network modulus $E_{f}^{0}$, (b) strain‐dependent fibril network modulus $E_{f}^{\varepsilon }$, (c) non‐fibrillar matrix modulus $E_{\text{nf}}$, (d) initial permeability $k_{0}$, (e) permeability strain‐dependency factor M, and (f) phase difference Δθ. Statistical significance is indicated by *‐markers (linear mixed model, *p < 0.05, **p < 0.01, ***p < 0.001).

**Figure 4:**
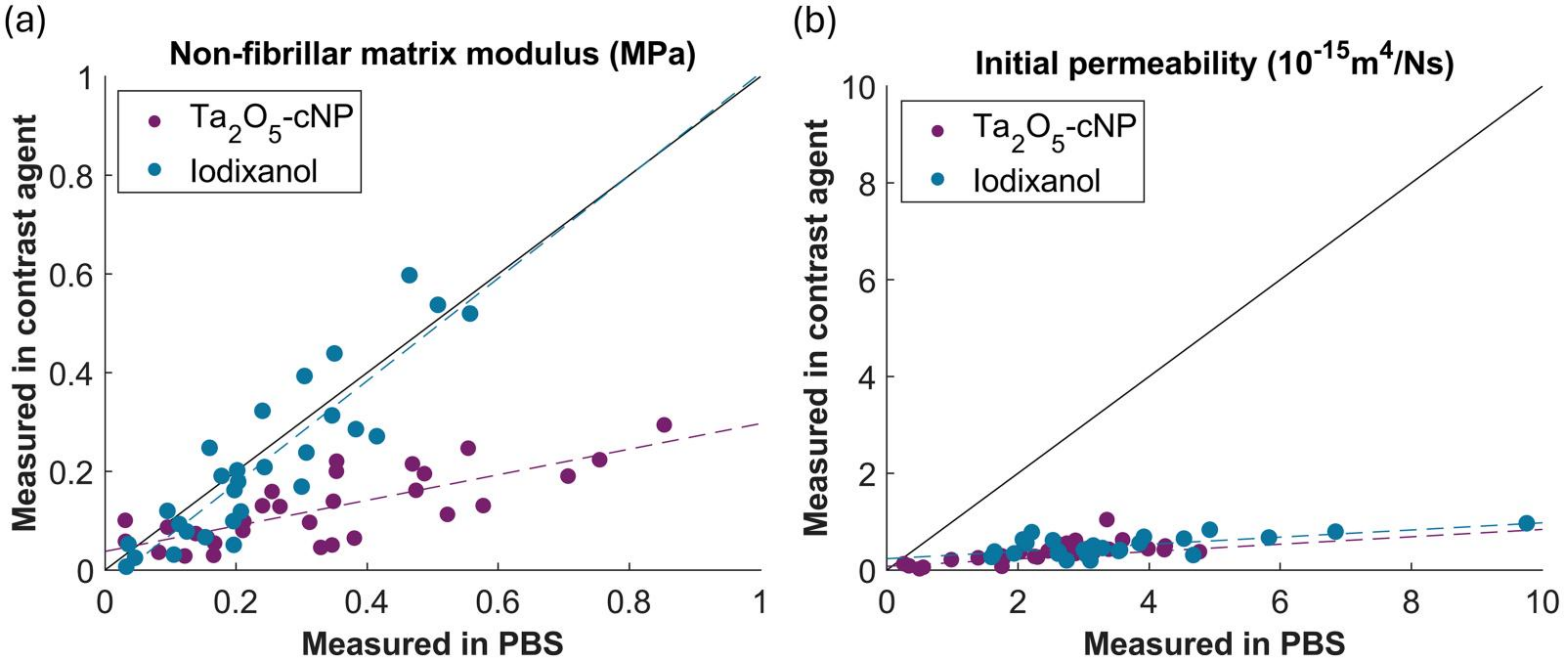
Behavior of samples after cationic tantalum oxide nanoparticle (Ta_2_O_5_‐cNP) and iodixanol immersion compared to measurement before contrast agent immersion in phosphate‐buffered saline (PBS). (a) Non‐fibrillar matrix modulus $E_{\text{nf}}$, and (b) initial permeability $k_{0}$. Straight line represents the one‐to‐one ratio, and dashed lines represent the linear fits to the data of before and after contrast agent immersion: $E_{\text{nf}}:\;0.259x+0.038$, and $k_{0}:\;0.120x+0.076$ for Ta_2_O_5_‐cNP, and $E_{\text{nf}}:\;1.040x-0.032$, and $k_{0}:\;0.074x+0.235$ for iodixanol.

Figure 5 shows differences in the mechanical behavior of cartilage before and after contrast agent immersion, as predicted by FE simulations. Ta_2_O_5_‐cNP was associated with a reduced response of cartilage across all steps (Figure 5a). Maximum IFLS increased significantly after Ta_2_O_5_‐cNP immersion in each peak compared to PBS peaks (*p* < 0.001, *p* < 0.001, *p* = 0.001, respectively, Supporting Information: Table S2). The mean IFLS was found to be higher before Ta_2_O_5_‐cNP and iodixanol only in the last stress‐relaxation step (*p* = 0.025, *p* = 0.009, respectively, Figure 5b, Supporting Information: Table S3).

**Figure 5:**
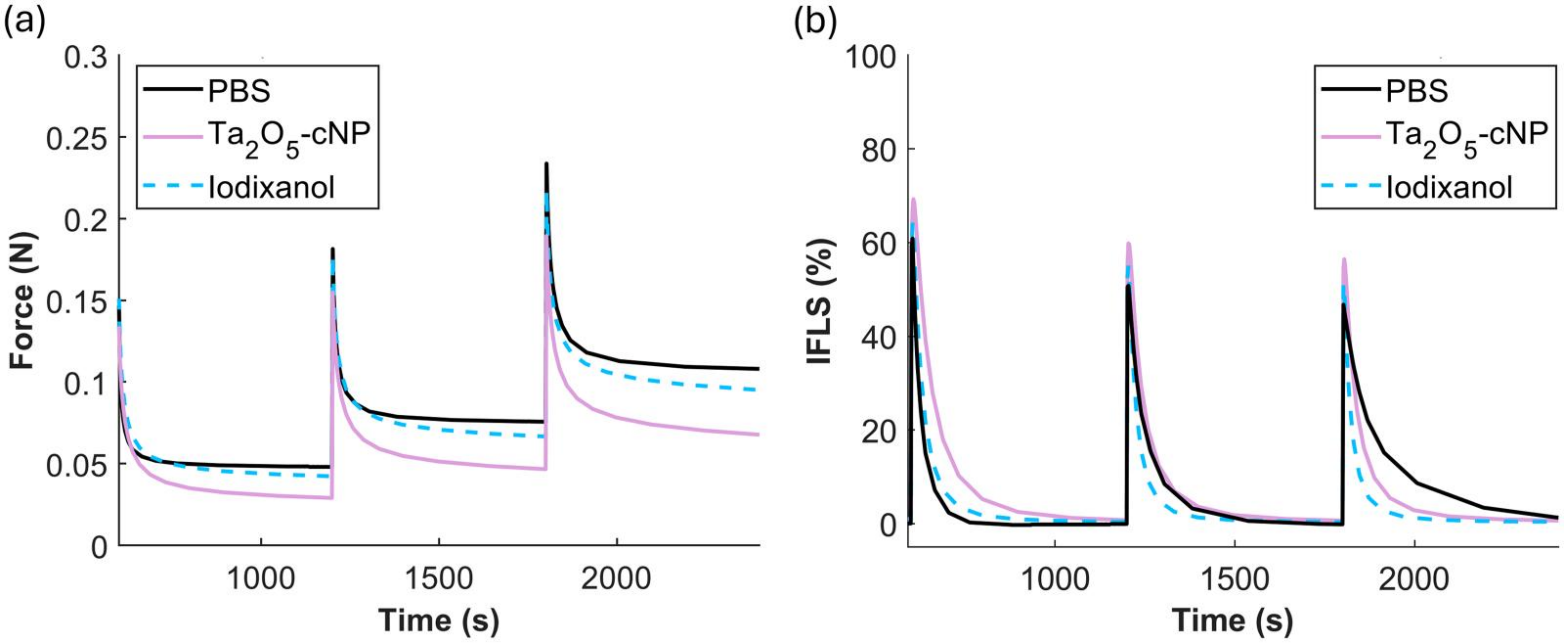
Finite element‐predicted response before and after contrast agent immersion. (a) Force‐time curves of simulated stress‐relaxation test, and corresponding (b) interstitial fluid support (IFLS) in phosphate‐buffered saline (PBS), cationic tantalum oxide nanoparticle (Ta_2_O_5_‐cNP) and iodixanol contrast agent.

### 3.2 Friction

The initial CoF was higher after Ta_2_O_5_‐cNP immersion compared to PBS (*p* = 0.012, Figure 6a), whereas no difference was observed at the equilibrium of friction measurement (Figure 6b). Conversely, the equilibrium CoF was lower after iodixanol immersion compared to PBS (*p* = 0.032, Figure 6b). Mean values and 95% CIs are presented in Supporting Information (Table S4).

**Figure 6:**
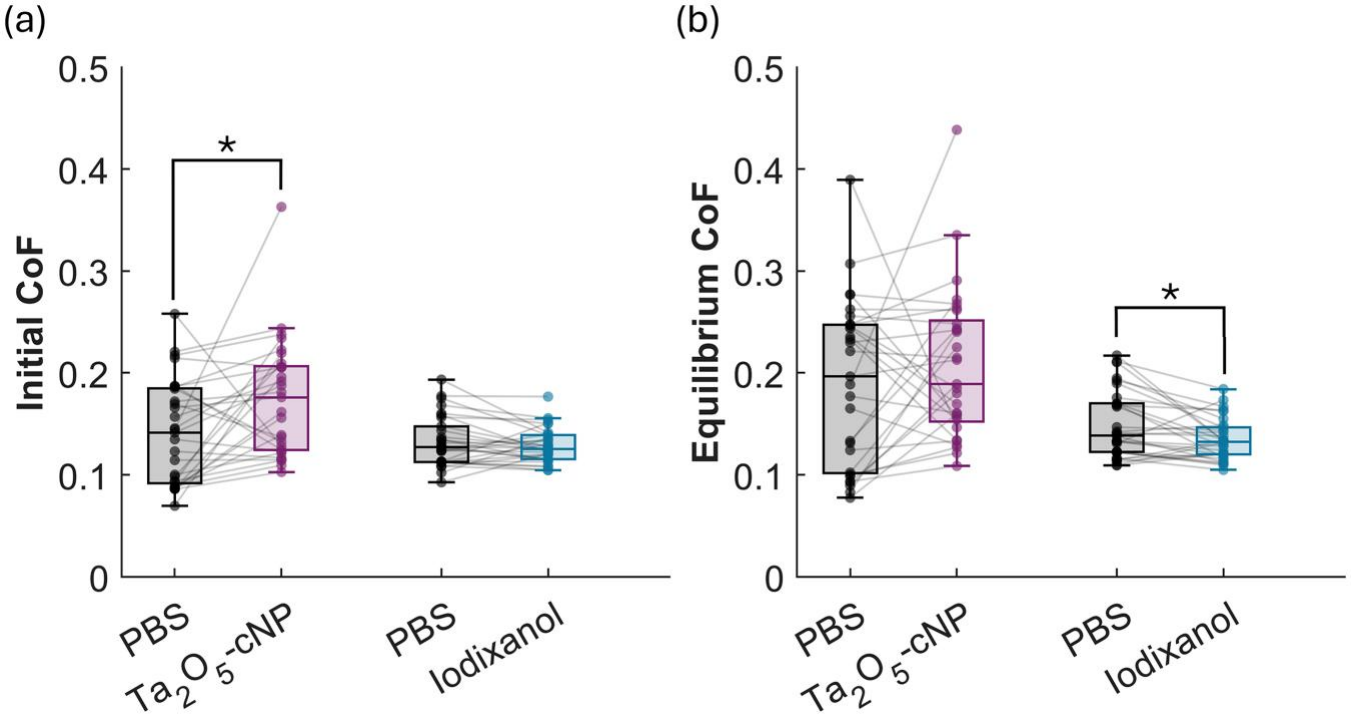
(a) Initial and (b) equilibrium coefficient of friction (CoF) of cartilage before (in phosphate‐buffered saline [PBS]) and after (in cationic tantalum oxide nanoparticle [Ta_2_O_5_‐cNP] or iodixanol) contrast agent immersion. Statistical significance (linear mixed model, *p* < 0.05) is indicated by *‐marker.

### 3.3 μCT Imaging

µCT measurements showed no change in cartilage thickness after contrast agent diffusion. Cartilage thickness was 1.6 mm (95% CI: 1.4, 1.7) prior to Ta_2_O_5_‐cNP immersion, and 1.5 mm (95% CI: 1.4, 1.7) after immersion (*p* = 0.674). Prior to iodixanol immersion, cartilage thickness was 1.2 mm (95% CI: 1.1, 1.4), and 1.3 mm (95% CI: 1.1, 1.4) after immersion (*p* = 0.665). At diffusion equilibrium, cartilage X‐ray attenuation was higher for Ta_2_O_5_‐cNP (93 h) than for iodixanol (38 h) (*p* = 0.034), with mean values of 1940 HU (95% CI: 1840, 2030) and 1790 HU (95% CI: 1700, 1890), respectively (Figure 7a). The mean maximum partition $P_{\max}$ was higher (*p* < 0.001) for Ta_2_O_5_‐cNP (478% [95% CI: 464, 492]) compared to iodixanol (46% [95% CI: 32, 60]) (Figure 7b). The diffusion time constant τ was greater (*p* < 0.001) for Ta_2_O_5_‐cNP (26.2 h [95% CI: 23.2, 29.2]) compared to iodixanol (1.8 h [95% CI: −1.3, 4.7]), indicating a slower progression to diffusion equilibrium.

**Figure 7:**
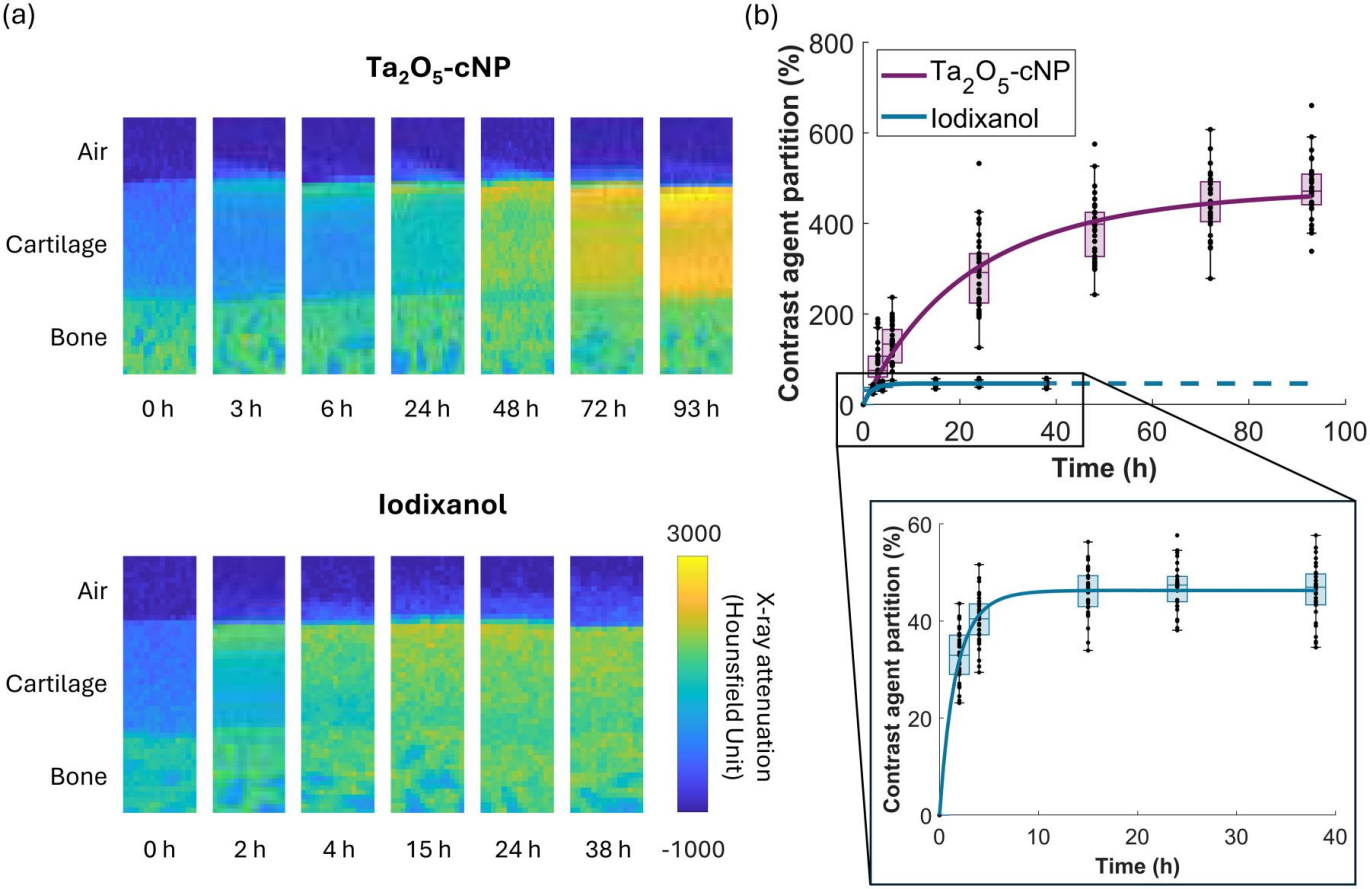
Contrast agent diffusion in cartilage assessed by microcomputed tomography (µCT). (a) µCT images of cartilage samples following immersion in cationic Ta_2_O_5_‐cNP (0–93 h) and neutral iodixanol (0–38 h), acquired in air at the indicated time points. (b) Bulk contrast agent partition (tissue attenuation normalized by bath attenuation) as a function of time. The exponential equation (Equation 2) of Ta_2_O_5_‐cNP (P(t)=478%×[1−exp(−t/26.2h)]) and iodixanol (P(t)=46%×[1−exp(−t/1.8h)]).

## 4 Discussion

The present findings demonstrate that contrast agents can influence both the elastic and fluid‐flow‐related behavior of articular cartilage. Ta_2_O_5_‐cNP was associated with reduced non‐fibrillar matrix modulus ($E_{\text{nf}}$) and increased maximum IFLS, whereas both agents were linked to decreased permeability ($k_{0}$, M) and phase difference (∆θ).

### 4.1 Effect of Ta_2_O_5_‐cNPs

Contrary to our first hypothesis, Ta_2_O_5_‐cNP contrast agent uptake caused a decrease in the non‐fibrillar matrix modulus $E_{\text{nf}}$ (Figure 3c), which describes the stiffness of AC created by negatively charged PGs [25, 38]. The observed softening of AC likely reflects a loss of FCD, resulting from the neutralization of PG negative charges by the cationic NPs. The softening of AC was similar in magnitude to that reported in study of Berni et al. [23], and to occur in the human tibial cartilage in early osteoarthritis [30]. For context, comparable changes have been reported in a rabbit ACLT model of early OA (~4 weeks post‐injury), with PG reductions of ~14%–38% and $E_{\text{nf}}$ decreases of up to ~50% [42]. However, in a physiological joint environment cartilage softening is expected to be less pronounced, as the Ta_2_O_5_‐cNP uptake would be limited to diffusion from the articulating surface. Any hypothesized reinforcement of the solid matrix by metal NP uptake was not quantifiable in our data. For that reason, our first hypothesis is rejected.

The maximum IFLS of each step in stress‐relaxation test was observed to increase after Ta_2_O_5_‐cNP immersion (Supporting Information: Table S2), despite the expected reduction in FCD due to charge neutralization. This increase may be associated with the concurrent decrease in permeability (Figure 3d,e), potentially reflecting hindered fluid transport within the matrix. This is also seen in the mean IFLS, as no similar strain‐dependent behavior was observed after contrast agent immersions compared to PBS (Figure 5b, Supporting Information: Table S3). Reduced fluid mobility would promote fluid pressurization and may also contribute to the observed decrease in phase difference ∆θ (Figure 3f), consistent with a reduced time‐dependent lag between stress and strain.

Ta_2_O_5_‐cNP contrast agent uptake resulted in moderate increase in friction at the initial state of the friction test (Figure 6a). The increase in initial CoF after the Ta_2_O_5_‐cNP immersion likely occurred because the nanoparticles, unlike larger ones used in an earlier study by Lawson et al. [33], were small enough (~3 nm) to diffuse into cartilage and interfere FCD–driven swelling pressurization. Since the testing involved short‐term unloading, which allows fluid to re‐enter the cartilage, IFLS played the dominant role during the whole measurement. This finding implies that ultrasmall nanoparticles may alter the fluid‐dependent frictional behavior of cartilage, especially during early loading. At the equilibrium of friction test, CoF did not differ significantly between conditions (Figure 6b). This likely reflects the fact that at equilibrium, interstitial fluid pressurization differences have fully dissipated, and effects of the contrast agents on fluid load support have diminished.

### 4.2 Effect of Iodixanol

Against our initial assumption of neutral iodixanol having minimal effect on cartilage function, exposure to iodixanol contrast agent had similar effect on viscous properties of AC than Ta_2_O_5_‐cNP. In early osteoarthritis, reduced GAG content has been associated with an increased phase difference, reflecting more viscous behavior [43]. In contrast, our results suggest that viscosity is also influenced by fluid transport, as iodixanol altered the viscous response without affecting tissue charge. Iodixanol uptake decreased initial permeability $k_{0}$, permeability strain‐dependency factor M, and phase difference ∆θ (Figure 3d–f). Similar to Ta_2_O_5_‐cNP, iodixanol likely reduces permeability by partially occluding cartilage pores, thereby limiting the strain‐dependent decrease in permeability (M). However, the maximum IFLS did not increase significantly after iodixanol immersion. This indicates that NPs have a greater impact on cartilages’ fluid flow than iodixanol, possibly due to larger size.

### 4.3 μCT Imaging

The absence of significant differences in $P_{\max}$ between MFC and DITG, and the lack of correlations with mechanical parameters, likely reflect the presence of unbound contrast agent within the samples. Unlike previous studies [20, 40, 44], diffusion was allowed from all surfaces rather than only through the articulating surface, increasing the proportion of freely distributed contrast agent that is not directly governed by cartilage composition. The contrast agent uptake of iodixanol was ~10 times lower than Ta_2_O_5_‐cNP, although the mass concentration of iodixanol was ~10 times higher than Ta_2_O_5_‐cNP. The obtained differences in diffusion are driven by the varied charge, size, and composition between the contrast agents [16, 20, 45, 46]. The diffusion of neutral contrast agents is mainly influenced by water content and matrix structure, while the diffusion of cationic contrast agents is driven by the electrostatic interactions with PGs [44]. Allowing diffusion from all sides likely accelerated the transport of Ta_2_O_5_‐cNPs (τ ~ 35% lower than in [20]) and increased the contribution of unbound agent. Despite the differences in transport dynamics, the maximum partition reached in this study (478%) remained within the range reported previously for equine cartilage (MFC: 704%–800%, DITG: 313%–372%) [20, 40], suggesting that while equilibrium levels were comparable, the pathway and distribution of contrast agent differed substantially.

In an earlier study, Tuppurainen et al. [40] showed using equine cartilage samples that dual‐contrast μCECT utilizing cationic Ta_2_O_5_‐NPs and a neutral iodixanol, can assess the FE model derived functional properties of cartilage. Ta_2_O_5_‐cNPs reflected the non‐fibrillar matrix properties, while iodixanol was indicative of permeability. In contrast, the present results suggest that these contrast agents also alter the functional properties of articular cartilage. Therefore, when using CECT to evaluate cartilage properties, the potential effects of contrast agent exposure should be considered and, where possible, accounted for in the interpretation of results. The observed linear decrease in non‐fibrillar matrix modulus and permeability after Ta_2_O_5_‐cNP immersion (Figure 4) suggests that it may still be possible to estimate initial cartilage condition prior to exposure. Neutralization of PGs due to Ta_2_O_5_‐cNP immersion represents a potentially significant limitation, and strategies to mitigate these effects should be considered particularly if such contrast agents are developed for clinical use.

### 4.4 Limitations

Several factors should be considered when interpreting the results. This study used bovine cartilage, which differs from human cartilage in aspects such as thickness, matrix composition, and permeability. These differences may influence the diffusion, retention, and mechanical impact of contrast agents. However, the core findings regarding charge‐ and size‐dependent effects on cartilage mechanics are likely to remain relevant across species [30]. The condition of the articular cartilage in the samples used in this study is not known, so the impact of any degeneration on the results cannot be evaluated. The iodixanol group samples showed slightly higher baseline values for $k_{0}$ and ∆θ, and slightly lower values for equilibrium CoF. However, the statistical analysis accounts for these differences, and key trends after exposure were consistent across both contrast agents. Therefore, the main conclusions remain valid. We also acknowledge that the study is limited by cartilage‐glass contact and rotational geometry in friction testing, which does not fully replicate physiological joint motion. However, the utilized measurement setups allow consistent measurement conditions. Biomechanical testing after contrast agent immersion was performed in the contrast agent instead of PBS to prevent potential leaking of contrast agent during the testing. Finally, one sample was excluded from the results regarding friction coefficients because of failed measurement, and two samples were excluded from the results regarding FE model parameters because the FE model could not converge, and therefore reliable material parameters could not be obtained. The model proved suitable for all other samples (Figure 2), justifying its utilization.

## 5 Conclusions

In summary, Ta_2_O_5_‐cNPs and neutral iodixanol contrast agents alter both the FCD and permeability of articular cartilage, thereby affecting its elastic and viscous properties. Cationic NPs reduced both cartilage stiffness and permeability, whereas the neutral molecular contrast agent primarily influenced fluid flow–related parameters. Notably, FE modeling enabled separation of the mechanical contributions of the non‐fibrillar matrix and interstitial fluid flow, providing insights which cannot be obtained with conventional mechanical testing alone. While the positive charge of the cationic NPs enhances their uptake and improves X‐ray visibility of cartilage, these physicochemical interactions also affect tissue mechanics. These findings emphasize the importance of carefully balancing particle charge and size in the development of contrast agents for cartilage imaging or therapeutic applications, particularly when targeting clinical translation.

## Author Contributions

Maria Rissanen contributed to research design, CT and biomechanical data acquisition, finite element modeling, data analysis and interpretation, and led the article drafting. Jiri Jäntti and Juuso Tuppurainen participated in research design, CT image acquisition, biomechanical data acquisition, data interpretation, and article revision. Milka Poimala synthesized and characterized the nanoparticles and revised the article. Brian D. Snyder, Mark W. Grinstaff, Mika E. Mononen, and Miitu K. M. Honkanen were involved in research design and article revision. Janne T. A. Mäkelä contributed to research design, data analysis and interpretation, and article drafting and revision. All authors reviewed and approved the final submitted article.

## Supporting information


Supporting File

## Data Availability

The data that support the findings of this study are available from the corresponding author upon reasonable request.
