# Supplementary material for: Cationic Tantalum Oxide Nanoparticle and Neutral Iodixanol Contrast Agents Alter Cartilage Mechanical Properties
Source: J Orthop Res. 2026 Oct 19;44(10):e70297. doi: 10.1002/jor.70297 (PMC13679883; doi:10.1002/jor.70297)
Supplement: Supplementary file 1 [file JOR-44-0-s001.pdf]

Supplementary material for

Cationic Tantalum Oxide Nanoparticle and Neutral Iodixanol Contrast Agents Alter Cartilage Mechanical Properties

Maria Rissanen[1,2], Jiri Jäntti[1,2], Juuso Tuppurainen[1,2], Milka Poimala[1,2], Brian D. Snyder[3], Mark W. Grinstaff[4], Mika E. Mononen[1], Miitu K. M. Honkanen[5], Janne T. A. Mäkelä[1,2]

[1]*Department of Technical Physics, University of Eastern Finland, Kuopio, Finland*

[2]*Diagnostic Imaging Center, Kuopio University Hospital, Wellbeing Services County of North Savo, Kuopio, Finland*

[3]*Boston Children's Hospital, Boston, MA, USA*

[4]*Departments of Biomedical Engineering, Chemistry, and Medicine, Boston University, Boston, MA, USA*

[5]*Center of Oncology, Kuopio University Hospital, Wellbeing Services County of North Savo, Kuopio, Finland*

Full contact details for the corresponding author: Maria Rissanen, Biophysics group, Department of Technical Physics, Faculty of Science, Forestry and Technology, University of Eastern Finland, Yliopistonranta 8, FI-70211 Kuopio, Finland, Email: maria.rissanen@uef.fi

Materials and Methods

Details on finite element modeling

A sample-specific axisymmetric FRPE finite element (FE) models were constructed using Abaqus (v6.22-1, Dassault Systèmes, Vèlizy-Villacoublay, France) to replicate the experimental stress-relaxation tests [1]. The total stress tensor is defined as the sum of the stresses arising from the fibrillar and non-fibrillar matrices, and the pore fluid pressure contribution [1–3]:

$$\boldsymbol{\sigma}_{\mathrm{t}} = \boldsymbol{\sigma}_{\mathrm{nf}} + \boldsymbol{\sigma}_{\mathrm{f}} - p\mathbf{I}, \tag{1}$$

where $\boldsymbol{\sigma}_{\mathrm{nf}}$ is the stress tensor of the non-fibrillar matrix, $\boldsymbol{\sigma}_{\mathrm{f}}$ is the stress tensor of the fibrillar matrix, p is the fluid pressure, and $\mathbf{I}$ is the unit tensor. The non-fibrillar stress tensor was modeled as Neo-Hookean hyperelastic material [3–6]:

$$\boldsymbol{\sigma}_{\mathrm{nf}} = \frac{1}{2}K_{\mathrm{nf}}(J - J^{-1})\mathbf{I} + \frac{G_{\mathrm{nf}}}{J}\left(\mathbf{F}\mathbf{F}^{\mathbf{T}} - J^{\frac{2}{3}}\mathbf{I}\right), \tag{2}$$

where K_{nf} is the bulk modulus of the non-fibrillar matrix, G_{nf} is the shear modulus, $\mathbf{F}$ is the deformation gradient tensor, and J is the determinant of the $\mathbf{F}$. The bulk and shear moduli can be expressed as a function of the non-fibrillar matrix modulus (E_{nf}) and the non-fibrillar matrix Poisson's ratio (fixed to $\nu_{\mathrm{nf}} = 0.42$ [2,4,7]) [6,8]:

$$K_{\mathrm{nf}} = \frac{E_{\mathrm{nf}}}{3(1 - 2\nu_{\mathrm{nf}})},$$
$$G_{\mathrm{nf}} = \frac{E_{\mathrm{nf}}}{2(1 + \nu_{\mathrm{nf}})}.$$

Fluid flow through the porous matrix is described by Darcy's law:

$$q = -k\nabla p, \tag{3}$$

where q is the rate of the fluid flow, k is the permeability of the material, and ∇p is the pressure gradient. Permeability's strain-dependency due to deformation in porous material can be taken into account by following [2,9–11]:

$$k = k_0\left(\frac{1+e}{1+e_0}\right)^{M}, \tag{4}$$

where k_0 is the initial permeability, M is constant describing the strain-dependency of permeability, and e and e_0 are current and initial void ratios, respectively. The initial void ratio

was set to 3.5, which corresponds to a fluid volume fraction of 78% [2,5,12]. The tensile stress of fibrillar network was modeled as nonlinear [2,4,13]:

$$\sigma_{\mathrm{f}} = \frac{1}{2} E_{\mathrm{f}}^{\varepsilon} \varepsilon_{\mathrm{f}}^{2} + E_{\mathrm{f}}^{0} \varepsilon_{\mathrm{f}}, \tag{5}$$

where σ_{f} and ε_{f} are stress and strain of the fibrillar component, respectively, E_{f}^{0} is the initial fibril network modulus and $E_{\mathrm{f}}^{\varepsilon}$ is the strain-dependent fibril network modulus. The collagen network was modeled using 4 primary fibrils oriented horizontally and 13 secondary fibrils distributed evenly in other directions. The relative density between primary and secondary fibrils was 12.16 [2,4,9,14].

FE model mesh consisted of 480 linear axisymmetric pore pressure continuum elements (element type CAX4P). FE mesh convergence was ensured by gradually reducing the number of elements from 1500 to 108. A mesh with 480 elements was decided to be used as it caused <1% difference in reaction force compared to a mesh with 1500 elements. Boundary conditions and displacement constraints were applied similarly to previous studies [4,5,8]: Contact between the flat-ended indenter and the cartilage was modeled impermeable, with a displacement boundary condition that restricted lateral movement and blocked fluid flow along the symmetry axis. To avoid mesh folding near the indentation edge, the indenter was modeled as an analytical rigid surface with frictionless hard contact, disallowing separation after contact. To mimic attachment to bone, the bottom of the cartilage was fixed in place, and fluid was not allowed to pass through this surface. Free flow (pore pressure = 0) was allowed through outer surfaces. To ensure that the mechanical material properties could be evaluated independently of tissue architecture, a homogeneous, depth-independent material was assumed, with constant fibrillar and non-fibrillar properties and void ratio throughout the tissue and an in-plane collagen fibril orientation, similarly as done previously [4,5,8,15].

Results

Table S1 Mean values and 95% confidence interval (CI) of constituent-specific finite element (FE) model parameters and phase difference ($\Delta\theta$) before (measured in phosphate-buffered saline [PBS]) and after (a) cationic tantalum oxide nanoparticle (Ta_2O_5-cNP), and (b) iodixanol immersion. Statistical significance (linear mixed model, $p < 0.05$) between values before and after contrast agent immersion is indicated by bold. E_f^0 is the initial fibril network modulus, E_f^ε is the strain-dependent fibril network modulus, E_{nf} is the non-fibrillar matrix modulus, k_0 is the initial permeability and M is the permeability strain-dependency factor.

**(a) Mean (95% CI)**	E_f^0 (MPa)	E_f^ε (MPa)	E_{nf} (MPa)	k_0 (10^{-15}·[m^4/Ns])	M	$\Delta\theta$ (degrees)
**PBS**	0.14 (0.10, 0.18)	10.81 (9.03, 12.60)	**0.33 (0.28, 0.39)**	**2.49 (2.18, 2.79)**	**16.95 (15.20, 18.69)**	**11.57 (10.45, 12.69)**
**Ta_2O_5-cNP**	0.10 (0.06, 0.14)	12.89 (11.10, 14.67)	**0.12 (0.07, 0.18)**	**0.38 (0.07, 0.68)**	**0.43 (-1.38, 2.23)**	**9.27 (8.15, 10.40)**
***p*-value**	$p = 0.179$	$p = 0.106$	**$p < 0.001$**	**$p < 0.001$**	**$p < 0.001$**	**$p < 0.001$**

**(b) Mean (95% CI)**	E_f^0 (MPa)	E_f^ε (MPa)	E_{nf} (MPa)	k_0 (10^{-15}·[m^4/Ns])	M	$\Delta\theta$ (degrees)
**PBS**	0.15 (0.11, 0.20)	9.89 (7.98, 11.81)	0.24 (0.18, 0.29)	**3.48 (3.03, 3.93)**	**15.59 (12.30, 18.07)**	**13.71 (12.72, 14.70)**
**Iodixanol**	0.14 (0.09, 0.19)	10.78 (8.87, 12.70)	0.21 (0.16, 0.27)	**0.52 (0.08, 0.97)**	**0.41 (-1.65, 2.47)**	**11.97 (10.98, 12.96)**
***p*-value**	$p = 0.747$	$p = 0.513$	$p = 0.556$	**$p < 0.001$**	**$p < 0.001$**	**$p = 0.016$**

Table S2 Mean values and 95% confidence interval (CI) of maximum interstitial fluid support (IFLS) after each compression in stress-relaxation experiment for all samples (first step excluded) before (measured in phosphate-buffered saline [PBS]) and after (a) cationic tantalum oxide nanoparticle (Ta_2O_5-cNP), and (b) iodixanol immersion. Statistical significance (linear mixed model, $p < 0.05$) between values before and after contrast agent immersion is indicated by bold.

**(a) Mean (95% CI) of maximum IFLS (%)**			
	**Step 2**	**Step 3**	**Step 4**
**PBS**	**62.95 (59.87, 66.02)**	**52.80 (50.66, 56.94)**	**50.44 (47.09, 53.79)**
**Ta_2O_5-cNP**	**70.95 (67.87, 74.02)**	**62.26 (59.12, 65.40)**	**58.48 (55.12, 61.83)**
***p*-value**	***p* < 0.001**	***p* < 0.001**	***p* = 0.001**

**(b) Mean (95% CI) of maximum IFLS (%)**			
	**Step 2**	**Step 3**	**Step 4**
**PBS**	62.29 (57.81, 67.16)	56.20 (52.75, 59.65)	52.47 (49.04, 55.90)
**Iodixanol**	67.31 (62.63, 71.98)	58.76 (55.31, 62.21)	54.94 (51.51, 58.37)
***p*-value**	$p = 0.149$	$p = 0.297$	$p = 0.311$

Table S3 Mean values and 95% confidence interval (CI) of interstitial fluid support (IFLS) of each stress-relaxation steps for all samples (first step excluded) before (measured in phosphate-buffered saline [PBS]) and after (a) cationic tantalum oxide nanoparticle (Ta_2O_5-cNP), and (b) iodixanol immersion. Statistical significance (linear mixed model, $p < 0.05$) between values before and after contrast agent immersion is indicated by bold.

**(a) Mean (95% CI) IFLS (%)**			
	**Step 2**	**Step 3**	**Step 4**
**PBS**	9.20 (4.44, 13.97)	12.73 (7.87, 17.58)	**16.98 (11.98, 21.98)**
**Ta_2O_5-cNP**	13.57 (8.80, 18.33)	10.38 (5.53, 15.24)	**8.83 (3.83, 11.84)**
***p*-value**	*p* = 0.200	*p* = 0.497	***p* = 0.025**

**(b) Mean (95% CI) IFLS (%)**			
	**Step 2**	**Step 3**	**Step 4**
**PBS**	6.88 (3.73, 10.03)	10.08 (6.16, 13.99)	**13.63 (9.08, 18.18)**
**Iodixanol**	7.52 (4.37, 10.66)	5.79 (1.87, 9.71)	**4.96 (0.42, 9.51)**
***p*-value**	*p* = 0.775	*p* = 0.126	***p* = 0.009**

Table S4 Mean values and 95% confidence interval (CI) of initial and equilibrium coefficient of friction (CoF) before (measured in phosphate-buffered saline [PBS]) and after (a) cationic tantalum oxide nanoparticle (Ta_2O_5-cNP), and (b) iodixanol immersion. Statistical significance (linear mixed model, $p < 0.05$) between values before and after contrast agent immersion is indicated by bold.

**(a) Mean (95% CI)**	**Initial CoF**	**Equilibrium CoF**
**PBS**	**0.14 (0.12, 0.16)**	0.19 (0.16, 0.21)
**Ta_2O_5-cNP**	**0.17 (0.15, 0.19)**	0.20 (0.18, 0.23)
***p*-value**	***p* = 0.012**	*p* = 0.352

**(b) Mean (95% CI)**	**Initial CoF**	**Equilibrium CoF**
**PBS**	0.13 (0.13, 0.14)	**0.15 (0.14, 0.16)**
**Iodixanol**	0.13 (0.12, 0.14)	**0.14 (0.13, 0.15)**
***p*-value**	*p* = 0.479	***p* = 0.032**

References

[1] M. Ebrahimi, M.A.J. Finnilä, A. Turkiewicz, M. Englund, S. Saarakkala, R.K. Korhonen, P. Tanska, Elastic, Dynamic Viscoelastic and Model-Derived Fibril-Reinforced Poroelastic Mechanical Properties of Normal and Osteoarthritic Human Femoral Condyle Cartilage, Ann Biomed Eng 49 (2021) 2622–2634. https://doi.org/10.1007/s10439-021-02838-4.

[2] J. Tuppurainen, P. Paakkari, J. Jäntti, M.T. Nissinen, M.C. Fugazzola, R. van Weeren, S. Ylisiurua, M.T. Nieminen, H. Kröger, B.D. Snyder, A. Joenathan, M.W. Grinstaff, H. Matikka, R.K. Korhonen, J.T.A. Mäkelä, Revealing Detailed Cartilage Function Through Nanoparticle Diffusion Imaging: A Computed Tomography & Finite Element Study, Ann Biomed Eng 52 (2024) 2584–2595. https://doi.org/10.1007/s10439-024-03552-7.

[3] W. Wilson, C.C. van Donkelaar, B. van Rietbergen, R. Huiskes, A fibril-reinforced poroviscoelastic swelling model for articular cartilage, Journal of Biomechanics 38 (2005) 1195–1204. https://doi.org/10.1016/j.jbiomech.2004.07.003.

[4] M. Ebrahimi, S. Ojanen, A. Mohammadi, M.A. Finnilä, A. Joukainen, H. Kröger, S. Saarakkala, R.K. Korhonen, P. Tanska, Elastic, Viscoelastic and Fibril-Reinforced Poroelastic Material Properties of Healthy and Osteoarthritic Human Tibial Cartilage, Ann Biomed Eng 47 (2019) 953–966. https://doi.org/10.1007/s10439-019-02213-4.

[5] M.T. Nissinen, N. Hänninen, M. Prakash, J.T.A. Mäkelä, M.J. Nissi, J. Töyräs, M.T. Nieminen, R.K. Korhonen, P. Tanska, Functional and structural properties of human patellar articular cartilage in osteoarthritis, Journal of Biomechanics 126 (2021) 110634. https://doi.org/10.1016/j.jbiomech.2021.110634.

[6] W. Wilson, C.C. van Donkelaar, J.M. Huyghe, A Comparison Between Mechano-Electrochemical and Biphasic Swelling Theories for Soft Hydrated Tissues, J Biomech Eng 127 (2005) 158–165. https://doi.org/10.1115/1.1835361.

[7] R.K. Korhonen, M.S. Laasanen, J. Töyräs, R. Lappalainen, H.J. Helminen, J.S. Jurvelin, Fibril reinforced poroelastic model predicts specifically mechanical behavior of normal, proteoglycan depleted and collagen degraded articular cartilage, Journal of Biomechanics 36 (2003) 1373–1379. https://doi.org/10.1016/S0021-9290(03)00069-1.

[8] J.T.A. Mäkelä, S.-K. Han, W. Herzog, R.K. Korhonen, Very early osteoarthritis changes sensitively fluid flow properties of articular cartilage, Journal of Biomechanics 48 (2015) 3369–3376. https://doi.org/10.1016/j.jbiomech.2015.06.010.

[9] P. Julkunen, P. Kiviranta, W. Wilson, J.S. Jurvelin, R.K. Korhonen, Characterization of articular cartilage by combining microscopic analysis with a fibril-reinforced finite-element model, Journal of Biomechanics 40 (2007) 1862–1870. https://doi.org/10.1016/j.jbiomech.2006.07.026.

[10] P. Julkunen, R.K. Korhonen, W. Herzog, J.S. Jurvelin, Uncertainties in indentation testing of articular cartilage: A fibril-reinforced poroviscoelastic study, Medical Engineering & Physics 30 (2008) 506–515. https://doi.org/10.1016/j.medengphy.2007.05.012.

[11] W.M. Lai, V.C. Mow, V. Roth, Effects of Nonlinear Strain-Dependent Permeability and Rate of Compression on the Stress Behavior of Articular Cartilage, J Biomech Eng 103 (1981) 61–66. https://doi.org/10.1115/1.3138261.

[12] P. Julkunen, T. Harjula, J. Iivarinen, J. Marjanen, K. Seppänen, T. Närhi, J. Arokoski, M.J. Lammi, P.A. Brama, J.S. Jurvelin, H.J. Helminen, Biomechanical, biochemical and structural correlations in immature and mature rabbit articular cartilage, Osteoarthritis and Cartilage 17 (2009) 1628–1638. https://doi.org/10.1016/j.joca.2009.07.002.

[13] L.P. Li, J. Soulhat, M.D. Buschmann, A. Shirazi-Adl, Nonlinear analysis of cartilage in unconfined ramp compression using a fibril reinforced poroelastic model, Clinical Biomechanics 14 (1999) 673–682. https://doi.org/10.1016/S0268-0033(99)00013-3.

[14] W. Wilson, C.C. van Donkelaar, B. van Rietbergen, K. Ito, R. Huiskes, Stresses in the local collagen network of articular cartilage: a poroviscoelastic fibril-reinforced finite element study, Journal of Biomechanics 37 (2004) 357–366. https://doi.org/10.1016/S0021-9290(03)00267-7.

[15] J.T.A. Mäkelä, M.R.J. Huttu, R.K. Korhonen, Structure–function relationships in osteoarthritic human hip joint articular cartilage, Osteoarthritis and Cartilage 20 (2012) 1268–1277. https://doi.org/10.1016/j.joca.2012.07.016.
